# Modelling the relationship between continuously measured glucose and electrocardiographic data in adults with type 1 diabetes mellitus

**DOI:** 10.1002/edm2.263

**Published:** 2021-05-29

**Authors:** Beatrice Charamba, Aaron Liew, Eileen Coen, John Newell, Timothy O’Brien, William Wijns, Andrew J. Simpkin

**Affiliations:** ^1^ School of Mathematics, Statistics and Applied Mathematics National University of Ireland Galway Galway Ireland; ^2^ Insight Centre for Data Analytics National University of Ireland Galway Galway Ireland; ^3^ Endocrinology Division Saolta University Healthcare Group Portiuncula University Hospital Galway Ireland; ^4^ Endocrinology Division Galway University Hospital Saolta University Healthcare Group Galway Ireland; ^5^ Regenerative Medicine Institute National University of Ireland Galway Galway Ireland; ^6^ The Lambe Institute for Translational Medicine, Curam and the Smart Sensors Lab National University of Ireland Galway Galway Ireland

**Keywords:** blood glucose self‐monitoring, diabetes mellitus, electrocardiography, type 1

## Abstract

**Introduction:**

Type 1 diabetes mellitus (T1DM) is associated with earlier onset of cardiovascular disease. Recent evidence has found hyperglycaemia appears to play a greater role in this association among T1DM compared to T2DM. This study investigates the relationship between glucose and QTc (a key cardiovascular measure) using data from continuous electrocardiogram (ECG) and glucose monitors.

**Methods:**

Seventeen adults with T1DM were recruited at a clinical facility in Ireland. A continuous glucose monitoring system was fitted to each participant that measured glucose every 5 min for 7 days. The participants simultaneously wore a vest with sensors to measure 12‐lead ECG data every 10 min for 7 days. Area under the glucose curve (AUC), proportion of time spent in hypoglycaemia and hyperglycaemia, and mean daily absolute deviation of glucose were calculated. Mixed effects ANOVA and functional regression models were fitted to the data to investigate the aggregate and time‐dependent association between glucose and QTc.

**Results:**

All participants were male with an average age of 52.5 (SD 3.8) years. Those with neuropathy had a significantly higher mean QTc compared to their counterparts. Mean QTc was significantly longer during hyperglycaemia. There was a significant positive association between QTc and time spent in hyperglycaemia. A negative association was found between QTc and time spent in hypoglycaemia. A functional model suggested a positive relationship between glucose and QTc at several times during the 7‐day follow‐up.

**Conclusion:**

This study used sensor technology to investigate, with high granularity, the temporal relationship between glucose and ECG data over one week. QTc was found to be longer on average during hyperglycaemia.

## Graphical abstract

Recent evidence has found hyperglycemia appears to play a greater role in this association among T1DM compared to T2DM. This study investigates the relationship between glucose and heartbeat (QTc) using data from 17 type 1 diabetics wearing continuous electrocardiogram (ECG) and glucose monitors for 1 week (i.e., 116,000 observations of glucose and ECG). QTc was found to be longer on average during hyperglycemia.

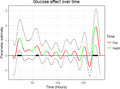

## 1 INTRODUCTION

Type 1 diabetes mellitus (T1DM) is associated with earlier onset of cardiovascular disease.^1^ Recent evidence has found hyperglycaemia appears to play a greater role in this association among T1DM compared to T2DM.^2^ Furthermore, young adults with T1DM can die overnight in their bed in what is called the ‘dead in bed syndrome’^3^ and hypoglycaemia has often been implicated as the possible cause.^4^ An irregular heartbeat and QT prolongation have been suggested as underlying mechanisms, which may result in ventricular arrhythmias, occurring during extended periods of physiological bradycardia.^5^ The relationship between glucose and heartbeat remains poorly understood, but technology which can measure both concurrently is now more economically viable for population health studies.^6^, ^7^

The prevalence of QT prolongation, which could range from 11% to 66.7% in people with T1DM, is more common in the presence of autonomic neuropathy.^8^, ^9^, ^10^ Hypoglycaemia has often been suspected of causing QT prolongation in people with T1DM. The Neuropathy Study Group of the Italian Society of the Study of Diabetes, Piemonte Affiliate, showed in a 5‐year cohort‐based prospective study that QT prolongation is predictive of an increased mortality in people with T1DM.^11^ Subsequently, Pickham et al.^12^ showed the potential link between hyperglycaemia and QT prolongation and that both are associated with increased odds of mortality in critically ill patients. It is currently unclear whether hyperglycaemia, and particularly, recurrent hyperglycaemia results in an increase likelihood of—and more—sustained QT prolongation.

This study used a 7‐day continuous data recording period to determine the relationship between QT and glucose adults with T1DM.

## 2 MATERIALS AND METHODS

### 2.1 Study design

After informed consent, all participants completed a questionnaire that included demographics, medications and medical history. Participants were fitted with a CGMS (iPro™ Professional Continuous Glucose Monitoring, Medtronic) which captured the glucose measurements, and these were retrieved at the end of the observation period. The participants also wore a vest with textile sensors to both record 12‐lead ECG data and send them to a secure cloud server (Master Caution device, HealthWatch). The vest and CGMS were worn for 1 week. The CGMS was blinded though ECG data were available on participants’ mobile phones during the study period. A participant diary was completed every day where they logged meals, medication, exercise, change of conducting hydrogel and change of vest. Self‐reported co‐morbidities were corroborated against medical records and by a physician. This study was approved by the Galway Clinical Research Ethics Committee.

#### 2.1.1 Inclusion criteria

1. Adult over the age of 18.
2. Consent to the study participation.
3. Presence of Type 1 Diabetes Mellitus.
4. Known fluctuations in blood sugar levels justifying monitoring.
5. Planned continuous blood sugar monitoring after the study.

#### 2.1.2 Exclusion criteria

1. Unable to consent to participate in the study.
2. Type 2 diabetes mellitus.
3. Not scheduled for routine continuous blood sugar monitoring.
4. Stable blood sugar levels for >3 months.
5. Unable to maintain clinical contact.
6. Allergy to silver.
7. Pregnancy.

### 2.2 Study variables

The data comprise both repeated and one‐time measurements. Glucose data were shifted 15 min backwards to adjust for the estimated time lag between blood and extracellular glucose that is measured by the CGMS guided by the results from other studies^13^, ^14^, ^15^ Glucose was coded as a categorical variable to allow for a simple comparison of ECG data across glucose groups: hypoglycaemia (<3.9 mmol/L), hyperglycaemia (>10 mmol/L) and normal glucose (3.9 mmol/L to 10 mmol/L inclusive) following similar studies.^16^

A hypoglycaemic episode was defined where an individual's glucose was below 3.9 mmol/L. The number of hypoglycaemic episodes was recorded for each patient during the 7 days. The average daily area under the glucose curve (AUC) per minute, average daily median absolute deviation (MAD) as a measure of glucose variability and mean proportion time spent in hypoglycaemia (<3.9 mmol/L), hyperglycaemia (>10 mmol/L) and normal glucose (3.9 mmol/L to 10 mmol/L inclusive) were calculated for each patient using the **MRCIEU/GLU** package in R. QTc was the outcome of interest from the ECG.

### 2.3 Study outcomes

All participants wore a vest with sensors that recorded a 12‐lead ECG and other measurements, including body temperature, respiratory rate, heart rate, respiratory amplitude, posture, QT, QRS, QTc, RR and PR intervals (Figure S1). The Master Caution device measured QT interval and corrected it for heartrate using Bazett's formula as the mean of the latest 16 RR intervals. These data were recorded every 10 min for 7 days, adding up to 1008 twelve lead ECG recordings per subject. The data had missing observations due to a change of vest or sensor gel renewal. Along with the vest, the CGMS collected glucose readings in mmol/L every 5 min for the same 7 days, adding up to 2016 glucose recordings per subject. All participants filled in a daily register where they recorded activities and any symptoms. Upon recruitment, demographic data were obtained for each participant, including age, sex, duration of diabetes, ethnicity, severe symptomatic hypoglycaemia in the previous year, smoking status, alcohol consumption, average insulin dosage, presence/absence of hypertension, retinopathy, neuropathy, dyslipidaemia and microalbuminuria. Severe hypoglycaemia was defined as any symptomatic hypoglycaemic event requiring assistance of another person to actively administer carbohydrate, glucagons, or other resuscitative actions and a glucose of <3.9 mmol/L. Neuropathy was defined based on clinical symptoms of neuropathy such as paraesthesia and clinical tests which confirm the diagnosis of neuropathy such as the positive 10 g monofilament test, absent or impaired vibration sense and reduced or absent ankle reflex.

### 2.4 Data cleaning

Data cleaning was performed on observations where both CGMS and ECG data were recorded. Expert opinion and relevant publications were used to determine a range of measurements that were not compatible with life, that is artefacts or signal loss. Realistic QTc interval was taken to be between 313 ms and 520 ms using bounds from a study by Pillai and Madhavan.^17^ The lower bound was taken to be the average minimum QTc‐3 standard deviations (377−3 × 21), while in subjects with neuropathy, the maximum was taken to be average maximum +3 standard deviations (439 + 3 × 26). There were no outlying observations in the CGMS data.

### 2.5 Statistical modelling

Background variables and study outcomes were summarized according to their nature, and mean and standard deviation were reported for continuous variables and frequencies and percentages for categorical variables. All analyses were carried out using R version 3.5.

After descriptive analysis, the data were analysed in three parts:

1. Comparing QTc across categories of co‐morbidities.
2. Modelling QTc against glucose variables.
3. Functional model of QTc and glucose.

#### 2.5.1 QTc and co‐morbidities

Mixed effects analysis of variance (ANOVA) models were used to investigate whether co‐morbidities (hypertension, retinopathy, neuropathy, smoking status, dyslipidaemia, severe hypoglycaemia and microalbuminuria) or glucose category were associated with QTc changes. The models compare the mean QTc in each category to verify the statistical significance of any differences. If there is a significant difference in mean values, it shows that a given factor is associated with QTc interval. The QTc data were recorded repeatedly over time in each individual, hence the data tend to be similar in each patient. To take into account any clustering effects, the subject variable was also included in the model as a *random effect*. This adjusts for within‐person correlation, but the interpretation remains the same as using standard regression methods.

#### 2.5.2 QTc and glucose summary variables

A regression model was fitted to determine the effect of number of glucose episodes, AUC and MAD on QTc controlling for baseline variables (age, hypertension, retinopathy, neuropathy, smoking status, dyslipidaemia, severe hypoglycaemia and microalbuminuria). These *functional* models take into account the time effect and within subject clustering.

#### 2.5.3 QTc and glucose over time

To fully utilize the data—that is continuous glucose and continuous QTc measured over time in the same individuals—more complicated statistical models are required. With both the main outcome (QTc) and exposure (glucose) measured at the same times (i.e., concurrent), the relationship between these variables also changes with time. A class of models called *functional regression models* is used to investigate the changing relationship between glucose and QTc. The result is a function over time which explains whether the relationship is positive (above 0) or negative (below 0). As with standard regression, there is a confidence interval which can be used for statistical significance testing and magnitude of effect. In the functional model, if the confidence interval is fully above 0, this suggests a significant positive relationship, and vice versa. The start time for each patient was set to midnight the day in which the patient was entered into the study. The time refers to the number of hours since the start of the study.

## 3 RESULTS

### 3.1 Descriptive statistics

**FIGURE 1:**
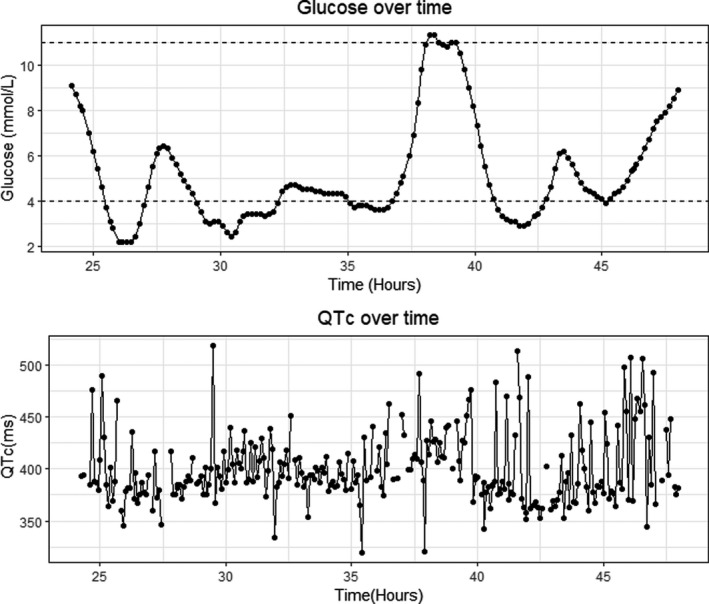
Glucose and QTc for one participant on the second day. The dots are actual measurements connected by a line

On cleaning the data, 9% of the QTc values were identified as outliers and removed. There were no missing glucose values. All participants were male with mean age 52.5 and average diabetes duration of 32.9 years as shown in Table 1. The average glucose was 10.2 mmol/L over the study period. During the study period, 14 of the participants experienced at least one hypoglycaemic episode, with a maximum of 14 episodes in one person. Daytime hypoglycaemia was seen in 11 participants with an average of 4 episodes. Nocturnal hypoglycaemia was experienced by 10 with an average of 2 episodes per person over the study period. The plots in Figure 1 show the glucose and QTc for one patient on a particular day. From the plot, glucose follows a smooth cyclic trajectory while QTc is irregular without any clear pattern.

**TABLE 1:** Participant summaries

Characteristic	*N *= 17
Age (Years) (mean, SD)	52.5 (13.2)
Duration of T1DM (Years) (mean, SD)	32.9 (11.3)
QTc (ms) (mean, SD)	415.1 (53.7)
Glucose (mmol/L) (mean, SD)	10.2 (1.1)
Mean daily AUC (mean, SD)	10.1 (1.8)
Mean daily percentage in hypoglycaemia (mean %, SD)	3 (3.6)
Mean daily percentage in hyperglycaemia (mean %, SD)	44 (18)
Mean daily percentage in normal glucose(mean %, SD)	53 (18)
Number of hypos (mean, SD)	4.5 (3.9)
Hypertension (*n*, %)	11 (65%)
Severe hypoglycaemia (*n*, %)	3 (18%)
Dyslipidaemia (*n*, %)	13 (76%)
Neuropathy (*n*, %)	4 (25%)
Microalbuminuria (*n*, %)	6 (35%)
Retinopathy (*n*, %)	11 (65%)
Smoker (*n*, %)	1 (5.8%)
Previous Smoker (*n*, %)	10 (58%)
Never Smoked	6 (35%)

The plots for mean QTc by glucose category and co‐morbidities (Figures S2 and S3) show that smokers, and subjects with neuropathy tend to have higher QTc. Subjects who previously experienced severe hypoglycaemia tend to have shorter QTc regardless of glucose category. Figures S4 and S5 show smoothed glucose and QTc by co‐morbidities over the study week. From the plots, mean glucose is similar with and without the presence of the co‐morbidities. However, mean QTc for neuropathy, retinopathy and dyslipidaemia subject was higher during the study week. Subjects who have experienced severe hypoglycaemia tend to have lower QTc.

### 3.2 Statistical modelling

#### 3.2.1 QTc and co‐morbidities

Table 2 shows the mean and standard deviation as well as the p‐value for the effect of co‐morbidities on QTc. There was a significant effect of neuropathy, which was associated with a 35 ms longer average QTc interval (CI: 33.1–36.4 ms). There was no significant association between QTc and hypertension, dyslipidaemia, microalbuminuria, severe hypoglycaemia or retinopathy. The mean QTc was slightly higher in hyperglycaemia with more variation in the normal glucose category. There seems to be little difference in QTc in hypoglycaemia and normal glucose category. Results from mixed models have shown there is a significant difference between mean QTc across glucose categories (Table 2A). From pairwise comparisons, QTc is significantly longer during hyperglycaemia compared to normal glucose and hypoglycaemia (9.97, *p*‐value = .0001) and (6.45, *p*‐value = .001), respectively. Mean QTc in hypoglycaemia was not significantly different from mean QTc in normal glucose (3.53, *p*‐value = .149) It can be noted from the pairwise comparisons that both hypoglycaemia and hyperglycaemia have longer mean QTc compared to normal glucose.

**TABLE 2:** Mean, standard deviation and ANOVA mixed model p‐value for QTc by and co‐morbidities and glucose category

Co‐morbidities Mean (SD)	Yes	No	*p*‐value
Severe Hypoglycaemia	394(40)	415(37.4)	.17
Hypertension	414(37.7)	412(36.9)	.96
Dyslipidaemia	413(37.5)	399(43.0)	.41
Retinopathy	413(36.6)	403(43.4)	.19
Microalbuminuria	409(44.6)	410(35.3)	.9
Neuropathy	440(38.2)	405(37.3)	.0076

**TABLE 2A:** Mean, standard deviation and ANOVA mixed model *p*‐value for QTc and glucose by glucose category

Glucose categories(mean)				
	Hypo	Normal	Hyper	*p*‐value
QTc (ms)	408.82	405.29	415.27	.0001
Glucose (mmol/L)	3.25	7.41	13.56	‐‐‐‐‐‐‐‐

#### 3.2.2 QTc and glucose summary variables

A functional regression model was fitted to determine the effect on QTc of MAD, AUC, number of hypoglycaemic episodes, mean proportion of time in hypoglycaemia and hyperglycaemia after controlling for baseline variables. Table 3 shows the parameter estimates and their confidence intervals. If the confidence interval contains zero, there is no statistically significant relationship between the glucose summary measure and QTc. From Table 3, there is evidence for a negative association between the number of hypoglycaemic episodes and QTc. However, looking at time spent in zones reveals more to this association. In particular, individuals with more time spent in hyperglycaemia had significantly longer mean QTc. There was no significant association between mean proportion time spent in hypoglycaemia and QTC. Mean daily AUC and MAD episode were not found to be associated with QTc.

**TABLE 3:** Functional regression of QTc against glucose summary variables

Glucose summary	Parameter estimate	Confidence interval
Number of hypos	−1.5318	−2.4465; −0.65
Mean AUC	0.27	−0.89;1.46
Mean Prop in hypo	−48.63	−148.96; 56.89
Mean Prop in hyper	23.39	1.85;45.79
MAD	1.65	−2.08; 5.34

#### 3.2.3 QTc and glucose over time

**FIGURE 2:**
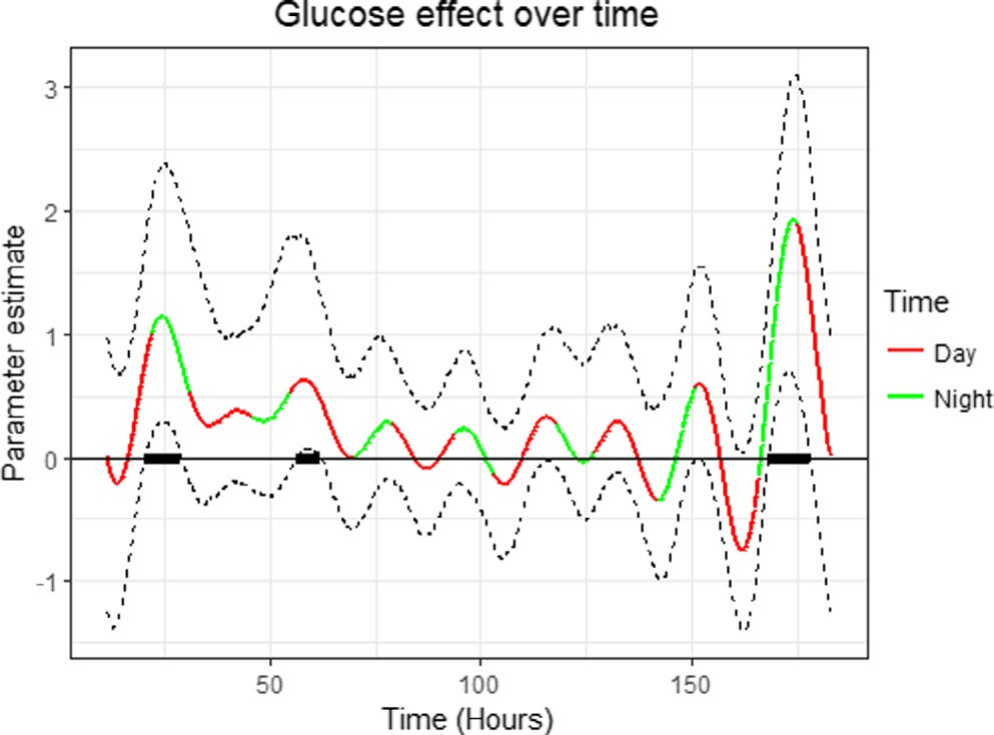
Parameter functions for the QTc against glucose coloured by time of day. Black bars show where there was a significant relationship

With continuously measured data over time, the effect of glucose on QTc can also change over time. Figure 2 shows the parameter function for the relationship between QTc and glucose colour‐coded by day (red) and night (green). From the plot, there is a non‐linear relationship between QTc and glucose which is mostly positive (i.e., higher glucose associated with longer QTc). There is evidence of a significant positive relationship between glucose and QTc where the (pointwise) confidence bands did not contain zero. For example, 56 h into the study, the parameter function is 0.6 ms (CI: 0.03–1.70 ms) implying that an increase of 1 mmol/L of glucose will result in an approximately 0.6 ms increase in mean QTc. That is a significant relationship since the confidence interval at that point does not include zero. Also, around 173 h the parameter function is 1.9 ms (CI: 0.7–3.0 ms). This is a significant relationship because the confidence interval is fully positive. There are three such intervals in the study week where a significant relationship between QTc and glucose was found. At these times, an increase in glucose was associated with an increase in mean QTc. There was no time interval in which there was a significant negative relationship between the two variables.

## 4 DISCUSSION

This study used continuously and concurrently measured data to investigate the association between extracellular glucose and QTc. We found that glucose has a positive relationship with QTc interval overall and at several specific times during the study. Intriguingly, it is notable that low blood glucose was not found to be associated with longer QTc interval though on average QTc was longer during hypoglycaemia compared to periods of normal glucose. The average QTc was negatively associated with number of hypoglycaemic episodes and mean proportion time in hypoglycaemia. The average QTc was negatively associated with the number of hypoglycaemic episodes, which is contrary to the findings from previous studies.^18^, ^19^, ^20^ As a matter of fact, the average QTc was positively associated with time spent in hyperglycaemia. Our observations confirm that QTc prolongation can be triggered by hyperglycaemia, rather than hypoglycaemia. This result supports Ninkovic et al.’s finding that hyperglycaemia and coronary heart disease are strong predictors of high‐risk QTc.^21^ In addition to these studies, Li et al. also confirm that high postprandial glucose level is a risk factor for QTc prolongation in T2DM patients.^22^

Our data show that neuropathy was the only significant correlation between QTc and diabetes related microvascular complication. This correlation was also seen by Vasheghani et al.,^23^ who demonstrated that mean QTc is high in participants with cardiac autonomic neuropathy compared to those without. It is well known that chronic hyperglycaemia results in neural damage,^24^ both sensory and autonomic. Our data showed that in the absence of neuropathy, the QTc values are the same as in controls. This further supports the theory that chronic hyperglycaemia results in neuropathy and that neuropathy is the cause of QTc prolongation.

Although our study is the first to model the concurrent relationship between glucose and QTc over a week, similar results were obtained by others ^12^, ^25^ who conducted cross‐sectional studies on critically ill patients. For instance, Pickham et al.^12^ found that patients with QTc longer than 440 ms have higher serum glucose levels. Lefrandt et al.^26^ found that high fasting glucose concentration level (>5 mmol/L) is predictive of QTc prolongation after adjusting for age, sex and cardiovascular risk factors. They included only healthy people in their study to determine the relationship between QTc duration, fasting glucose and cardiovascular risk indicators, for example blood pressure, cholesterol, body mass index etc. In determining the prevalence and risk factors for prolonged QT interval and QT dispersion in people with type 2 diabetes mellitus, Ninkovic et al.^21^ found that hyperglycaemia and coronary heart disease were strong predictors of high‐risk QTc (>500 ms). The results of this study support the theory that hyperglycaemia appears to have a more profound effect on cardiovascular risk in T1DM than T2DM.^2^

Others have found longer average QTc in diabetics who smoke,^27^ an observation we could not verify because our data included just one smoker. There was no significant association between hypoglycaemia and QTc which is in line with Christensen et al.^18^ who concluded that it was not clear whether more pronounced hypoglycaemic could affect QTc. Their study findings support the theory that an underlying cardiac disease must be present for hypoglycaemia to cause lethal cardiac arrhythmia.

In contrast to our study, Tsujimoto et al.^28^ concluded that people with type 1 and type 2 diabetes mellitus with severe hypoglycaemia experienced many critical problems that could lead to cardiovascular disease. While they found significant associations between severe hypoglycaemia and hypertension, they did not see a significant association with QTc prolongation. Gill et al.^29^ who also used CGM and continuous ECG found that QTc prolongation was evident during hypoglycaemia in people with T1DM. Their analysis did not consider the repeated nature of the data, and they used simple t tests to compare average QTc in hypoglycaemic versus hyperglycaemic periods. Gruden et al.^20^ did a cross‐sectional study where ECG was measured, and patients were asked whether they have severe hypoglycaemic events. They found that those who experienced severe hypoglycaemia are more likely to have prolonged QTc (>400 ms). In their study, Ireland et al.^30^ found that QTc was longer during hypoglycaemia compared to euglycaemia, which was also seen in this study though not significant. A meta‐analysis from Fitzpatrick et al.^31^ also found a relationship between hypoglycaemia and QTc prolongation across 15 studies. This association was found in T1DM participants which is contrary to the findings presented here. Most of these studies included in this meta‐analysis were based on cross‐sectional data.

The main strength of this study was the availability of concurrently and continuously measured QTc and glucose for 7 days. Hence, data were more representative than cross‐sectional data that do not reflect the diurnal variation. In addition, our analyses used all the available data, which gives better results compared to those methods using summary measures for analysis. Furthermore, to minimize clinical heterogeneity, we only included T1DM male participants with no previous cardiovascular events, though studies have shown that in the presence of neuropathy, the absolute cardiovascular disease risk in men and women was equal.^32^ Previous studies showed that QTc prolongation is prevalent in people with T2DM, ranging from 15.4% to 67%, and hypoglycaemia has often been implicated especially in the presence of cardiovascular disease.^21^, ^22^, ^33^, ^34^, ^35^

The statistical modelling presented here allows for a changing relationship between QTc and glucose, and we have found times when there were associations and others were there were none. Although we cannot draw specific conclusions about the timing of these associations, our study shows that there are associations, and we focus on their direction and magnitude. A limitation of our study was that the available vest would only fit males, and no females were recruited and formally, we cannot generalize our findings to the whole population of people with T1DM. Another potential limitation could be that some medications taken by participants could have prolonged QTc. This is not likely because the cardiac medications included anti‐arrhythmic drugs, anticoagulants, lipid lowering drugs, beta blockers, ACE/ARB, and none are prone to cause QTc prolongation.

Our finding on the positive relationship between hyperglycaemia and QTc in people with T1DM is intriguing and merits further studies with longer and larger sample size. It also further supports the current guidelines which recommend achieving the optimal percentage of time in range in this population. Our study also highlights the potential clinical importance of the use of the continuous and concomitant monitoring of glucose and electrocardiograph in routine clinical settings. This study is one of the first to use sensor technology to investigate the temporal relationship between glucose and ECG data over one week with such high data density. Continuously and concomitantly measured multiple data sets increase the strength of studies to find such associations and similar studies should be undertaken to increase our understanding of this complex, dynamic relationship. In conclusion, we found associations which suggest a positive relationship between hyperglycaemia and QTc in people with T1DM.

## CONFLICT OF INTEREST

No competing interests existed.

## AUTHOR CONTRIBUTIONS

**T.O** and **A.L** involved in conceptualization (equal), review and editing (equal) and original draft preparation (supporting). **W.W** involved in conceptualization (lead), project administration (lead), funding acquisition (lead) and review and editing (supporting). **E.C** involved in investigation (lead). **A**.**S** involved in methodology (lead), original draft preparation (supporting), review and editing (equal) and fund acquisition (supporting). **J.N** involved in review and editing (supporting). **B.C** involved in methodology (equal), formal analysis (lead), original draft preparation (lead), and review and editing (lead).

## Supporting information

Supplementary Material

## Data Availability

The data that support the findings of this study are available on request from the corresponding author. The data are not publicly available due to privacy restrictions.
