# Supplementary material for: Modelling the relationship between continuously measured glucose and electrocardiographic data in adults with type 1 diabetes mellitus
Source: Endocrinol Diabetes Metab. 2021 May 29;4(3):e00263. doi: 10.1002/edm2.263 (PMC8279601; doi:10.1002/edm2.263)
Supplement: Supplementary file 1 — Supplementary Material [file EDM2-4-e00263-s001.docx]

# Supplemental material


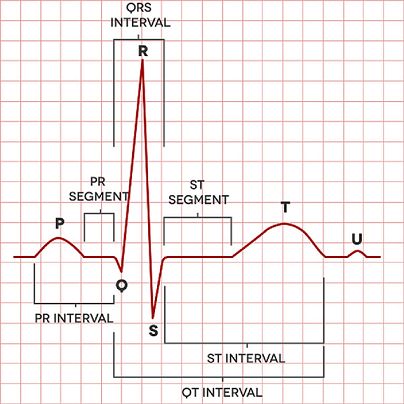


*Figure S1: ECG tracing for one heartbeat (Image from www.123rf.com)*


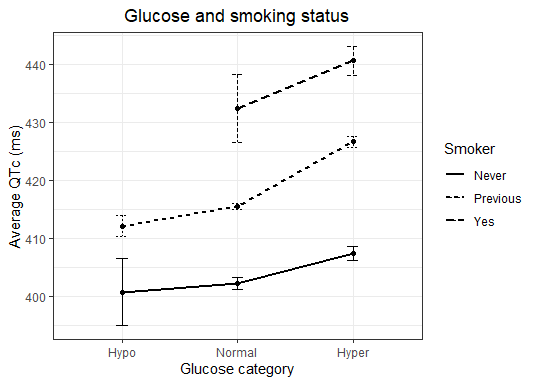


Figure S2: Mean QTC by glucose category and smoking status. Solid line indicates those who never smoked, dotted line previous smokers and broken lines smokers.


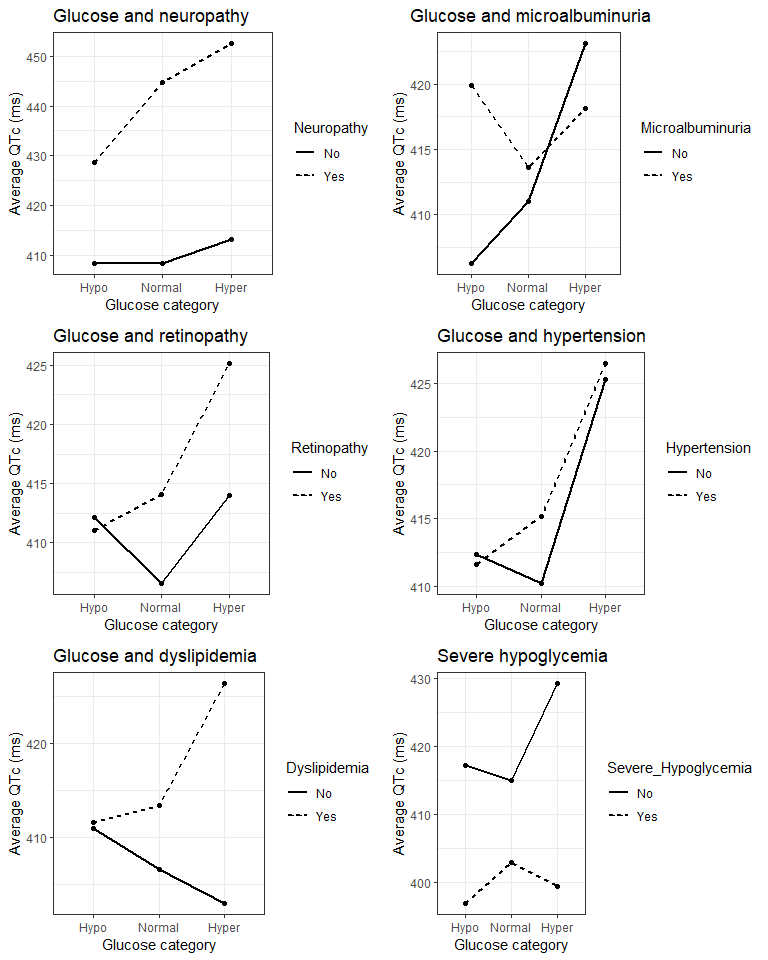


Figure S3: Average QTC by glucose and medical condition. Solid lines indicate absence of condition and broken lines indicate presence of condition


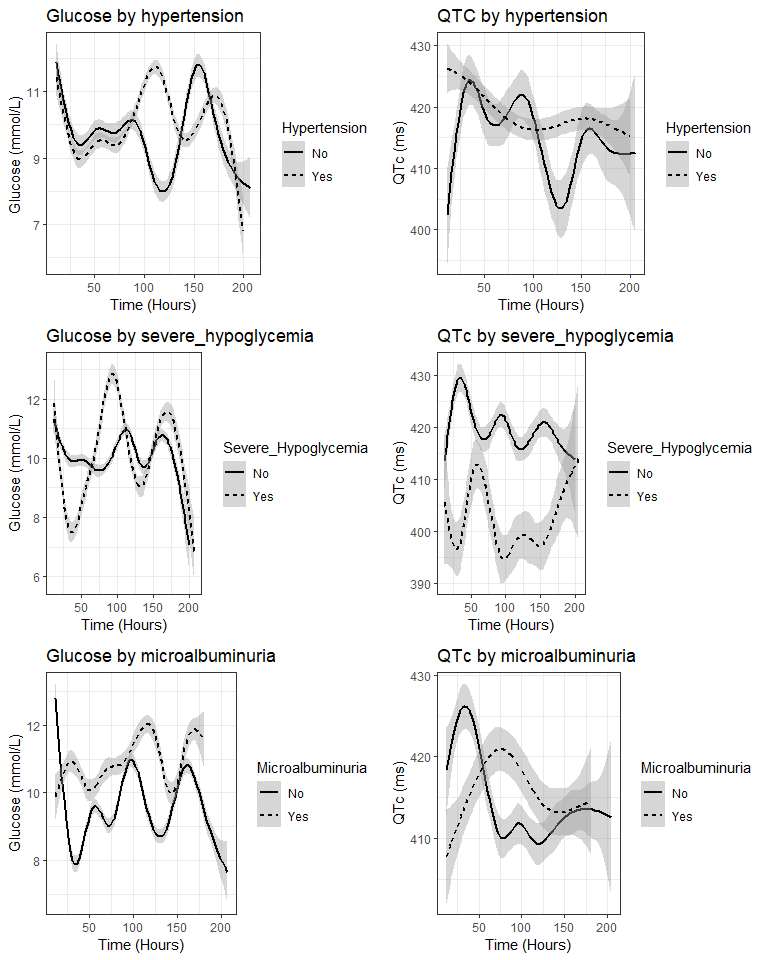


Figure S4: Smoothed QTC and glucose over time by medical condition. Solid lines indicate absence of condition and broken lines indicate presence of condition.


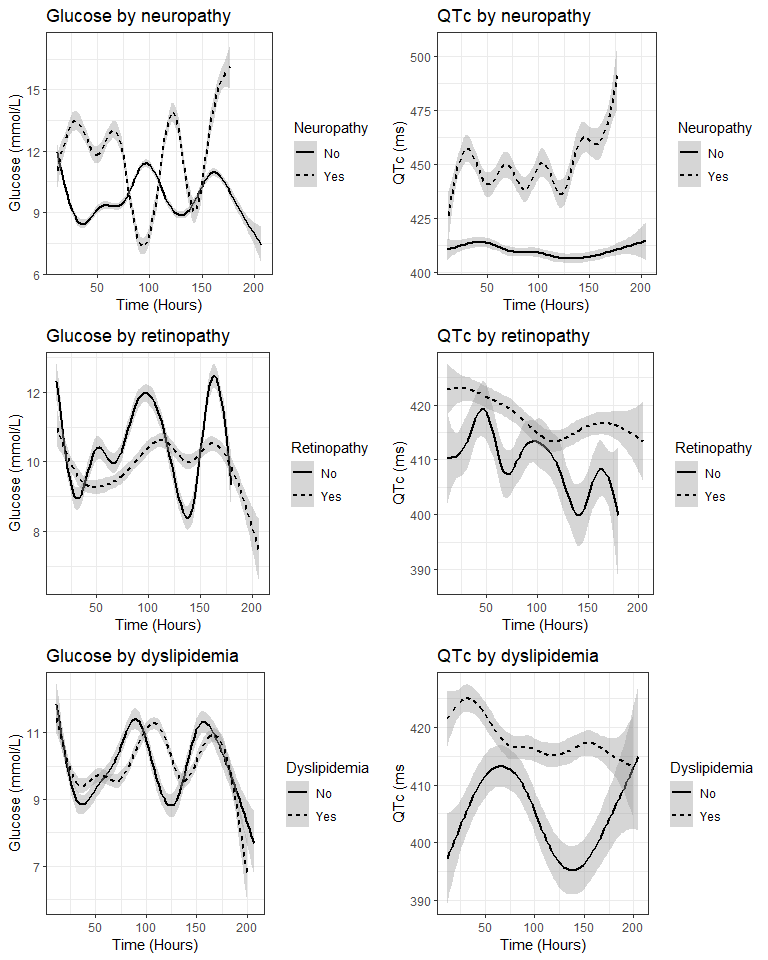


Figure S5: Smoothed QTc and glucose over time by medical conditions. Solid lines indicate absence of condition and broken lines indicate presence of condition.
